# Anthropogenic disturbance affects calling and collective behaviour in corvid roosts

**DOI:** 10.1098/rstb.2023.0185

**Published:** 2024-06-23

**Authors:** Hannah R. Broad, Alex J. Dibnah, Anna E. Smith, Alex Thornton

**Affiliations:** ^1^ Centre for Ecology and Conservation, University of Exeter, Penryn TR10 9FE, UK; ^2^ Centre for Ecosystem Science, School of Biological, Earth and Environmental Sciences, University of New South Wales, Sydney, 2052 NSW, Australia

**Keywords:** anthropogenic disturbance, collective behaviour, sleep, acoustic communication, cognition, corvids

## Abstract

Acoustic communication plays an important role in coordinating group dynamics and collective movements across a range of taxa. However, anthropogenic disturbance can inhibit the production or reception of acoustic signals. Here, we investigate the effects of noise and light pollution on the calling and collective behaviour of wild jackdaws (*Corvus monedula*), a highly social corvid species that uses vocalizations to coordinate collective movements at winter roosting sites. Using audio and video monitoring of roosts in areas with differing degrees of urbanization, we evaluate the influence of anthropogenic disturbance on vocalizations and collective movements. We found that when levels of background noise were higher, jackdaws took longer to settle following arrival at the roost in the evening and also called more during the night, suggesting that human disturbance may cause sleep disruption. High levels of overnight calling were, in turn, linked to disruption of vocal consensus decision-making and less cohesive group departures in the morning. These results raise the possibility that, by affecting cognitive and perceptual processes, human activities may interfere with animals’ ability to coordinate collective behaviour. Understanding links between anthropogenic disturbance, communication, cognition and collective behaviour must be an important research priority in our increasingly urbanized world.

This article is part of the theme issue ‘The power of sound: unravelling how acoustic communication shapes group dynamics’.

## 1. Introduction

Communication between individuals can have crucial impacts on survival and reproductive success [1]. Acoustic signals, such as vocalizations, can be a particularly efficient form of communication, as they can propagate over large distances and remain effective even when other sensory modalities are obscured, or individuals are dispersed [2,3]. One common function of acoustic communication is its use in coordinating group dynamics and facilitating collective behaviour. To reap the benefits of group living, such as predator avoidance, increased foraging efficiency and access to mates [4,5], social groups require decision-making processes to maintain cohesive movements [6,7]. There is increasing evidence that consensus decisions and group movements are mediated by acoustic communication across taxa, including mammals [2,6,8–11], birds [3,12–14] and insects [15,16]. For instance, groups of meerkats (*Suricata suricatta* [6]) and African wild dogs (*Lycaon pictus* [10]) reach consensus to move between foraging patches or hunting grounds when a certain ‘quorum threshold’ of individuals are calling, while in birds, decisions to leave roosting sites *en masse* are triggered by a growing crescendo of callers [13,17].

Despite growing appreciation of the ecological value of collective behaviour [18] and the role of vocal mechanisms in underpinning group coordination, we know little about how these mechanisms could be disrupted by anthropogenic disturbance, such as noise and light pollution. The expansion of urban areas over the past century has led to a vast increase in anthropogenic disturbance [19–22], and may have profound ecological implications for animal populations, with such disturbances being linked to a variety of behavioural, physiological and fitness impacts [19,23–25].

Noise and light pollution are classed as sensory pollutants, meaning they alter the ability of animals to assess and interact with their environment [26]. For animals that rely on acoustic signals, these sensory pollutants may inhibit the ability to receive, process and respond to acoustic stimuli [27–30]. First, noise pollution may affect the fidelity of acoustic signals by altering the soundscape and impeding the propagation of signals through the environment, a phenomenon known as masking [27,29–31]. Chronic noise pollution can also induce hearing damage and loss, reducing an individual’s capability to receive, process and use acoustic signals [29,32,33]. Alternatively, anthropogenic disturbance may act as a cognitive distraction, reallocating an individual’s attention away from relevant stimuli and preventing an efficient response [34,35]. Such impacts can have substantial fitness consequences by negatively affecting individuals’ ability to assess the acoustic environment, detect prey or predators [36,37] and communicate with conspecifics [27,31,38,39]. For example, lab and field experiments on great tits (*Parus major*) showed that noise pollution impacts the production and perception of alarm calls, with individuals adjusting the amplitude of their calls and failing to respond to conspecific calls in the presence of traffic noise playbacks [37].

Anthropogenic disturbance may impair vocal communication indirectly by inhibiting cognitive performance [28,29]. First, noise and light pollution may affect individuals’ stress responses [40–43]. High chronic levels of stress hormones are thought to have detrimental long-term effects on brain development and cognition [44–46]. Therefore, if chronic noise or light is perceived as a stressor, this could negatively impact cognitive function [29,43]. For example, tree swallow (*Tachycineta bicolor*) nestlings exposed to artificial light at night [40] and great tits roosting under white light pollution [47] showed increased levels of the stress hormone corticosterone. Additionally, chronic light conditions inhibited spatial memory and cognitive performance in house crows (*Corvus splendens* [46]). There is also evidence that individual cognitive phenotypes may themselves affect responses to vocalizations. In wild Western Australian magpies (*Gymnorhina tibicen dorsalis*), for example, individuals that performed better in tests of associative learning showed greater maintenance of anti-predator responses to alarm calls in the presence of anthropogenic noise [48].

Links between anthropogenic disturbance, cognition and collective behaviour may be particularly important because urban pollutants may negatively alter cognitive performance by causing sleep disturbance. Artificial light at night alters natural cycles of light and darkness [49,50], disrupting the circadian rhythms that help to regulate cognitive performance [51,52]. Circadian rhythms, sleep and light are inherently linked, meaning light pollution can impact the sleep–wake cycle [53,54]. Furthermore, noise pollution is increasingly occurring throughout the night, which has historically been a quiet period [55]. Noise pollution can disrupt sleep cycles, reducing the quality or length of sleep [56–58]. Sleep is an important and widespread behaviour in animals [59] that is thought to serve many essential roles [53], including maintenance of brain health and function [60–62]. Indeed, many cognitive abilities are thought to be sustained by sleep [60,63,64], including some aspects of acoustic signalling, such as audio discrimination and memory consolidation [51,60,65,66]. Sleep disturbance could, therefore, have profound effects on cognition and communication. There are growing efforts to understand the impacts of anthropogenic disturbance on sleep and cognition through studies of captive animals [56,57,67], although the majority of research to date has been conducted on humans [68–74]. Therefore, the extent to which anthropogenic disturbance causes sleep disruption and resultant cognitive impacts in wild animals is still unknown [75].

If our understanding of the impacts of noise and light pollution (e.g. masking, sleep disturbance and reduced cognitive performance) on individual behaviour is in its infancy, even less is known about the impacts of these factors on social interactions, in particular, the coordination of collective behaviour [76,77]. There is some evidence that anthropogenic noise and light disrupt collective behaviour in fish [77–79]. For example, playbacks of anthropogenic noise (piledriving) reduced the cohesiveness of shoaling sea bass *(Dicentrarchus labrax* [77]). However, to our knowledge, no study has focussed on the effects of anthropogenic disturbance on acoustic communication in the context of collective behaviour. Impacts on cohesion could have negative effects on predation risk and information use, potentially undermining certain benefits of group living [77,80]. As global urbanization continues to rise [81] and anthropogenic disturbance associated with these urban areas becomes more widespread [21,49], it is vital that we better understand the impacts of anthropogenic disturbance on acoustic communication and group dynamics, especially in wild species.

In this study, we investigate the effects of noise and light pollution on vocal communication and collective behaviour of wild jackdaws (*Corvus monedula*). Jackdaws, a highly social corvid species, are an ideal study system for this research as they use vocalizations to coordinate collective behaviour (anti-predator mobbing [82,83]; collective movements [13]), and are widespread across both urban [84–88] and rural areas [89]. Jackdaws form winter roosts that can comprise up to several thousand birds [13,89]. Upon arrival at dusk, they make loud, distinctive calls that gradually decrease as they settle down to sleep for the night ([89]; see also ‘Audio Recordings’ in electronic supplementary material). In the early mornings, jackdaws become increasingly vocal again prior to a sudden mass departure [13]. Observational and experimental evidence indicates that jackdaws vocalize to signal their readiness to depart from roost sites. Steep increases in calling up to a high level of intensity trigger a group-level consensus, leading to mass collective departures [13]. If, however, jackdaws’ ability to reach a consensus becomes disrupted, they risk losing the fitness benefits of departing in a cohesive group. An understanding of these effects is therefore crucial for informing conservation and management strategies [19,90]. Although jackdaws are not currently of conservation concern [91], insights into the impacts of anthropogenic disturbance on communication and collective movements may benefit the conservation and management of the many other species that exhibit collective behaviour.

Using overnight audio recordings and videos of departures across jackdaw roost sites with varying exposure to anthropogenic disturbance (i.e. artificial light and noise produced by human activities), we aimed to evaluate the effects of noise and light pollution on calling activity and group movements. We made five key predictions. As arrivals at the roost in the evening are accompanied by calling, and birds may call in response to anthropogenic disturbance [92,93], we predicted (i) that jackdaws would take longer to settle down and cease calling when arriving at more disturbed roost sites. We also predicted (ii) that greater night-time disturbance would lead the birds to call more during the night, suggesting disruption of sleep [94,95]. If disruption of rest or sleep interferes with the perception of and responses to vocal signals, we predicted (iii) that the birds would show shallower rates of increase in calling in the morning (indicating a lack of consensus [13]) when they had experienced greater levels of disruption overnight. As a consequence, we predicted that greater overnight disruption would be linked to (iv) reduced temporal coordination of departures (i.e. less collective, measured as the proportion of roost members leaving together in the largest departing group [13]) such that (v) roosts would take longer to empty in the morning.

## 2. Methods

### (a) Roosting sites

**Figure 1.**
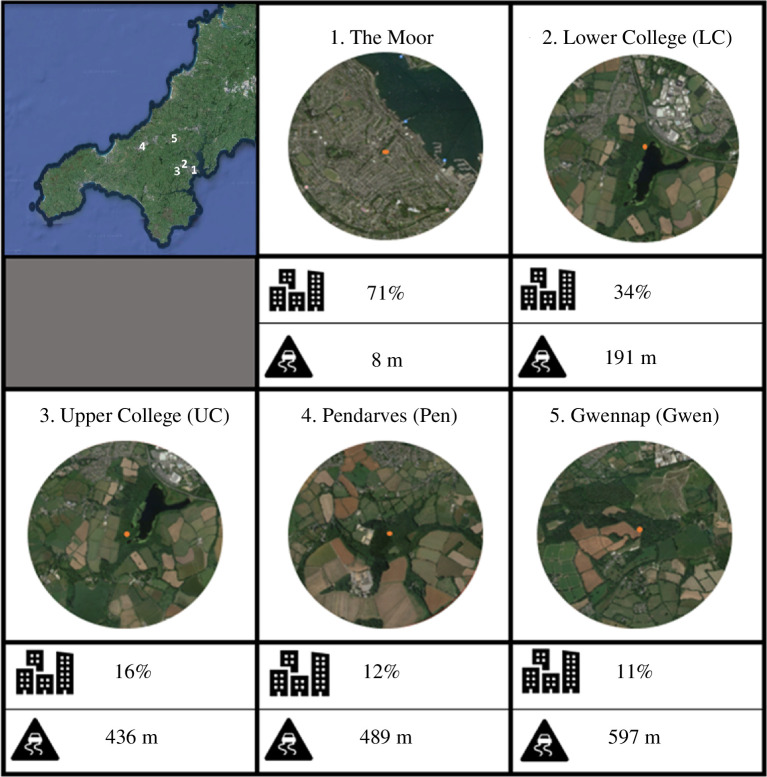
From top left to bottom right – A map of Cornwall showing the location of the five roost sites, followed by 1km radius images around each roost (from Google Earth): (1) The Moor (located in the centre of Falmouth, next to busy pubs and a bus stop); (2) Lower College (LC; located near a busy A-road and an industrial estate); (3) Upper College (UC; more rural, but in the vicinity of a busy A-road and a residential area); (4) Pendarves (Pen; rural, but near some quiet country lanes) and; (5) Gwennap (Gwen; rural location with no nearby roads or residential areas). The icons represent the percentage of the 1 km radius that is urban and distance to the nearest classified road. Numbers next to roost names correspond to locations on the map.

We studied five jackdaw roost sites across rural and urban locations in Cornwall, UK, during the winter roosting season between November 2021 and March 2022 (see electronic supplementary material for details of roost site selection). The roost sites varied in levels of urbanization from a site adjacent to busy pubs and a bus stop in the centre of the town of Falmouth (The Moor) to more remote sites in the countryside (Gwennap). The percentage of urban land use in a 1 km radius around roosts ranged from 11% to 71% (figure 1), as determined through QGIS analysis [96] using land use data collected from the NERC Environmental Information Data Centre [97]. The distance to the closest classified road (determined by www.findmystreet.com, excluding small country lanes) ranged from 8 to 597 m (figure 1).

### (b) Data collection

#### (i) Audio recordings

We deployed between two and four Open Acoustic AudioMoth loggers, depending on the size and shape of roosts, at each site in two week intervals throughout the roosting season, collecting audio recordings between 16:00 (before birds arrived at the roost in the evening) and 08:00 (after they departed in the morning). Following pilot observations to determine where jackdaws tended to settle within the roosts, we secured the audio loggers to trees approximately 20 m apart within central roost locations at a height of approximately 2.5 m (see electronic supplementary material for further details). To avoid disturbing roosting birds, we deployed and collected the audio recorders during the day. Across the season, we collected 329 nights of audio recordings across the five sites. Nights when audio recorders failed or when high levels of wind and/or rain occurred were excluded from analyses. In total, we deemed 157 nights of audio were of sufficient quality across the five sites (Upper College/UC = 22, Pendarves/Pen = 32, The Moor = 38, Gwennap/Gwen = 33 and Lower College/LC = 32).

#### (ii) Roost observations

We used video recordings to estimate temporal coordination of departures. We arrived approximately 1 h before sunrise and filmed departures using a Panasonic Lumix DMC-FZ200 camera from a standardized position at least 50 m from the roost site to avoid disturbance, remaining at the roost until no more jackdaws could be seen. Excluding cases where equipment failed or poor conditions (e.g. low light or heavy rain) impeded filming, we collected 29 successful departure videos (see electronic supplementary material for more details).

#### (iii) Meteorological data

##### Light levels

Light was recorded using custom-made loggers consisting of Raspberry Pi zero microcomputers [98] as a base, a TCS34725 Flora Colour Sensor [99] and a TSL2591 HDR digital light sensor [100]. It was not possible to place loggers at the tops of trees where jackdaws were roosting, so to capture ambient light conditions in the area, we placed one light logger at each site within 50 m of central roosting trees but away from canopy cover during periods of audio recording. Measurements were taken every 15 min between 16:00 and 08:00. Equipment failure at our most urban site meant we were only able to capture light levels at the four other sites. We collected 120 nightly averages of overnight light levels across the four sites. We averaged light data (lux) between 16:00 and 08:00 to give a measurement of overnight light levels for each night of audio recording.

##### Weather conditions

As weather conditions could influence patterns of activity and calling, we obtained daily rainfall and average windspeed records from local Met Office weather stations (see electronic supplementary material for more details). We collated daily mean windspeed and rain data for all data points (*n* = 168). We excluded data if there was heavy rain, as this masked jackdaw calls in our analyses. Any rain in our dataset was therefore at very low levels and was converted to a simple binary ‘1’ (presence of rain within 24 h) or ‘0’ (no presence of rain within 24 h; see electronic supplementary material for more details). We measured temperature at each site every 5 min for 24 h a day during the two-week audio recording periods using Tiny tags Plus 2 (TGP-4505) data loggers deployed in the same trees alongside the audio recorders. We collected 128 nightly averages of temperature across five sites. We used the period between 16:00 and 08:00 to calculate an average nightly temperature, measured in °C.

### (c) Data processing

#### (i) Acoustic analysis of jackdaw vocalizations

We processed audio recordings in Audacity (www.audacityteam.org). As jackdaws would often move between trees within the roost throughout the night or across the roosting season, we compared the audio recordings collected on the same nights from different recording locations and selected the recording that collected the clearest jackdaw calls (i.e. the recording with the least noise disturbance from wind or background noise and the loudest jackdaw calls). Once we had selected the appropriate audio recording for each night, we removed the period of time before the first arrival of birds and after the last departure. To limit potential impacts of background noise, we then applied 6 dB of noise reduction to each night’s recording using a 5 min noise profile taken between 02:00 and 04:00 in the absence of jackdaw calls or noises whose frequencies overlap with those of jackdaw calls. This reduced the interference of low-frequency background noise in our acoustic analysis of jackdaw vocalizations, while avoiding any noise reduction of jackdaw calls.

To quantify jackdaw calling activity throughout each night, we used spectral analyses in Matlab [101] to calculate power spectral density (PSD) values, following Dibnah *et al*. [13]. PSD values measure the power (in dB) of each frequency component (Hz) for each second of audio. To capture jackdaw calling, we selected a frequency range of 1000–3000Hz (electronic supplementary material, figure S1). Although jackdaw calls can occasionally exceed this frequency range, these values sufficiently capture calling while reducing representation of non-jackdaw noises of similar frequencies (see electronic supplementary material for more detail).

To avoid the inclusion of calling before the arrival of birds to the roost or after their departure (i.e. as they fly to or from the site), data before the highest PSD value in the first 2 h of the recording and after the highest PSD value in the last 2.5 h of the recording were removed. We then normalized PSD (nPSD) values for each night to account for variations in the position of audio recorders relative to the position of birds in the roost.

##### Prediction 1

To calculate the rate at which jackdaws settle down and cease calling after arriving at roosts, we conducted linear regressions each night using the first hour of nPSD values against time (s). We selected an hour based on observations of jackdaw arrivals and audio spectrograms viewed in Audacity across the five sites. We obtained regression slopes as a measure of rate of settling upon arrival in normalized dB Hz^−1^ h^−1^. On the majority of evenings (147/157), slopes were negative, reflecting the gradual cessation of calling, but in 10 instances, slopes were slightly positive as the birds did not quieten over the course of an hour post arrival.

##### Prediction 2

As overnight activity has been linked to sleep deprivation in roosting birds [94], we used levels of overnight calling as an indicator of night-time disturbance. By listening to audio recordings and comparing spectrograms with associated nPSD values, we determined that nPSD values above a threshold of 0.2 captured night-time jackdaw calls while excluding background noise (electronic supplementary material, figure S2). We therefore calculated the proportion of each night that jackdaws spent calling as the proportion of seconds during which nPSD values were ≥0.2, covering the period after the birds settled down post-arrival until 1.5 h before they departed in the morning. As anthropogenic sounds could occasionally exceed our 0.2 threshold—particularly at The Moor, our most urban site—we also tested the robustness of results by re-running analysis using a more conservative threshold of 0.4, which minimizes any impacts of anthropogenic noise, but at the cost of excluding some jackdaw calls (see electronic supplementary material for more details).

##### Prediction 3

Following Dibnah *et al*. [13], we calculated the rate at which jackdaw calling increased prior to roost departures, using linear regressions of the last hour and a half of PSD values against time (s). A period of one-and-a-half hours was selected based on observations of jackdaw departures and audio spectrograms across the five sites. Regression slopes provided a measure of rate of increasing calling intensity prior to departure in normalized dB Hz^−1^ h^−1^.

### (ii) Video analysis of departures

#### Prediction 4

To estimate the temporal coordination of departing birds and the total number of jackdaws at each roost site (hereafter ‘roost size’), we reviewed all departure videos frame-by-frame. We defined a group departure as when two or more birds flew away from the roost at the same time within the same time window (i.e. 10 s), in the same direction and were separated by two or more seconds from prior or subsequent departing groups (see electronic supplementary material for details). An independent coder who was blind to study predictions and site analysed a random selection of 10% of the roost videos to verify video analysis. Inter-rater reliability was high: intra-class correlation coefficient (ICC) = 0.99 (*p* < 0.001, confidence interval 0.87–1.00). Roost size ranged from a mean of 92 jackdaws at the smallest roost to 1473 at the largest. Therefore, the smallest possible group (i.e. two birds departing together) could constitute between 0.14% and 2.17% of the total roost.

#### Prediction 5

We also recorded the time taken for roosts to empty. This was calculated as the time from the first jackdaw(s) leaving the roost until the last departure from the roost (i.e. until no more jackdaws were present).

### (iii) Acoustic analysis of background noise

To determine levels of background noise for each night, we extracted three periods of audio: the hour before the birds arrived, 2 h during the night (23:00–01:00) and the hour before continuous departure calling began (identified by visual inspection of spectrograms for each night). To reduce any possible interference from jackdaw calls, we extracted a noise profile from a period of frequent jackdaw calling during the arrival and used this to apply 6 dB of noise reduction to the background noise profile. We then calculated PSD values to quantify noise intensity as described in §2c(i), but covering a wider frequency range of 0–4000Hz. This frequency range was determined by reviewing frequency analysis plots of periods with extensive anthropogenic noise disturbance when jackdaws were not present at our most urban site and is consistent with frequency ranges for anthropogenic noise pollution used in previous studies ([102,103]; see electronic supplementary material for more details). We then normalized PSD values and calculated mean nPSDs per time period per site as measures of local background noise levels before arrival, during the night and prior to departure. We calculated these values for every night of roost audio recording and departure video bar one in our dataset (*n* = 167), which was excluded due to audio recording failure.

### (d) Statistical analysis

We carried out statistical analyses in R v. 4.2.2 [104]. To test our predictions, we conducted linear mixed model analyses using the package lme4 [105]. For each model, we checked residual plots for violation of model assumptions (using the package dharma [106]) and tested for multicollinearity between predictors by assessing the variance inflation factor (VIF). The response variables were the rate of decline in calling post-arrival (arrival analysis: Prediction 1), the proportion of overnight calling (overnight analysis: Prediction 2) and for morning departure analyses, the rate of increase in calling intensity prior to departure (Prediction 3), the temporal coordination of departures (Prediction 4) and the time taken for the roost to empty (Prediction 5). All models included roost site and month as random effects to account for temporal and spatial non-independence. Proportion of night calling was transformed using a logit transformation to fit model assumptions.

Roost site-level measures of the proportion of urban area within a 1 km radius, distance to the nearest road and artificial brightness (collected from www.lightpollutionmap.info) were highly correlated with each other and moderately correlated with our measures of noise disturbance (electronic supplementary material, table S1). To examine the potential effects of anthropogenic disturbance, we therefore focused on nightly noise disturbance, as this was measured on a finer scale with variability within, as well as between, sites. Our key predictors were the levels of background noise before arrivals (Prediction 1), overnight (Prediction 2) or in the hour prior to departure (Predictions 3, 4 and 5). As collective decision-making during departures could be impacted both by direct effects of noise (e.g. through masking calls) and by knock-on effects of being disturbed throughout the night, departure analyses also included levels of overnight calling as an additional predictor. The total number of birds in the roost (roost size) and daily weather conditions (mean temperature, mean windspeed and whether any rain fell) were also included as explanatory variables. As our light level meter at The Moor failed, we ran additional analyses restricted to the other four sites to determine whether light levels predicted settling post-arrival, overnight calling, and the increase in calling intensity before departure. This additional analysis also allowed us to test whether effects still held when excluding The Moor, as this highly urban site could be driving effects. Limited sample sizes precluded including light intensity (and thus, excluding data from The Moor) in analyses of the cohesiveness of departures and time for roosts to empty. All explanatory variables in our models were scaled to allow for comparisons of the effect size of predictors. We present the effects of every variable in full model selection tables in the electronic supplementary material, where the importance of each predictor was determined by dropping individual factors from a full model (using the drop1 function from the R package lme4 [105]) and assessing if this significantly decreased the AIC of the model using likelihood ratio tests.

We had 121 roost audio recordings available for audio analysis with corresponding data for all predictor variables when all sites were included in analysis. Analyses, when The Moor was excluded (so overnight light levels could be included), used 81 nights of audio for which corresponding data were available for all other predictors. Although all 29 successful departure videos were assessed, only 17 of these had associated estimates of proportion of night calling owing to high rain or wind disturbance on the audio recording of the previous night. The datasets supporting this article have been uploaded in the Figshare digital repository (doi: 10.6084/m9.figshare.25661085; [107]). All plots were created using the package ggplot2 [108].

## 3. Results

### (a) Arrival analysis

#### (i) Prediction 1

**Figure 2.**
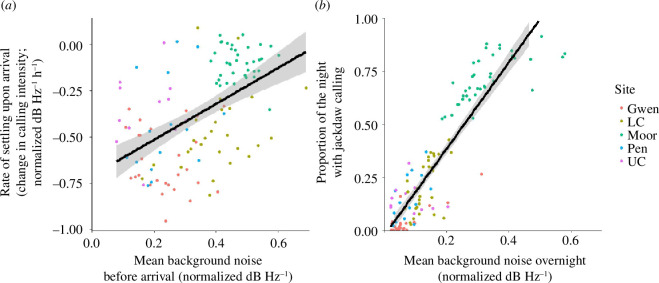
The influence of (*a*) mean background noise on the rate at which jackdaws reduce calling after arrival at the roost (lower values represent a steeper rate of decline in calling following arrival, indicating that jackdaws settle more quickly); and (*b*) mean overnight background noise on the proportion of the night that jackdaws spent calling. Dot colours indicate the different roost sites and the regression lines with standard errors are displayed.

As predicted, jackdaws took longer to settle upon arrival at the roost when levels of background noise were higher. When all sites were included in the model, we found shallower rates of decline in calling (i.e. flatter slopes, indicating jackdaws were taking longer to settle) when levels of background noise in the previous hour were higher (estimate (s.e.) = 0.208 (0.098); *χ* ^2^ = 4.823, d.f. = 1, *p* = 0.028; figure 2*a*). No other terms were significant in this model (electronic supplementary material, table S2). However, the effect of background noise did not hold when The Moor was excluded from the analysis. Here the only significant variable was roost size, with roosts with larger numbers of jackdaws becoming quiet more quickly (estimate (s.e.) = −0.342 (0.173); *χ* ^2^ = 5.281, d.f. = 1, *p* = 0.022; electronic supplementary material, table S3), although this effect must be interpreted with caution as it is difficult to disentangle effects of roost size from other, unmeasured site-level effects (e.g. the roost size area and distribution of jackdaws within the roost) that could influence rates of settling.

### (b) Overnight analysis

#### (i) Prediction 2

When all sites were included in the model, we found that, as predicted, jackdaws spent a greater proportion of the night calling when overnight levels of background noise were higher (estimate (s.e.) = 0.408 (0.063); *χ* ^2^ = 38.147, d.f. = 1, *p* < 0.001; figure 2*b*). Overnight calling was also higher when temperatures were lower and windspeed was higher (electronic supplementary material, table S4), although these weather effects were not robust if The Moor was excluded (electronic supplementary material, table S5). However, when The Moor was excluded, we still found a strong effect of background noise (estimate (s.e.) = 0.360 (0.067); *χ* ^2^ = 27.730, d.f. = 1, *p* < 0.001). There was also some evidence in this analysis that overnight calling was lower when light levels were higher (estimate (s.e.) = −0.371 (0.091); *χ* ^2^ = 16.278, d.f. = 1, *p* < 0.001) and when roost sizes were larger (estimate (s.e.) = −0.383 (0.111); *χ* ^2^ = 6.406, d.f. = 1, *p* = 0.011).

We found the same effects of noise levels on overnight calling if we used a more conservative threshold of nPSD = 0.4 to classify when jackdaw calls occurred in both analyses, including and excluding The Moor (*p* ≤ 0.001; see electronic supplementary material for more details).

### (c) Departure analysis

#### (i) Predictions 3–5

Contrary to our predictions, we found no evidence that noise levels were directly associated with group departures. Instead, the establishment of vocal consensus and the coordination of departures were negatively related to levels of overnight calling.

##### Prediction 3

**Figure 3.**
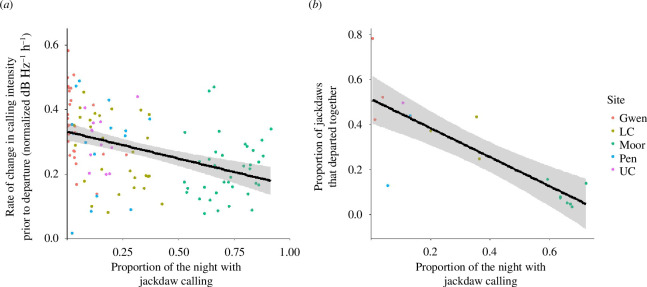
The influence of the proportion of the night that jackdaws spent calling on (*a*) the rate at which jackdaws increase calling prior to departure from roosts and (*b*) the temporal coordination of jackdaw groups departing collectively from roosts. Dot colours indicate the different roost sites and the regression lines with standard errors are displayed.

When all sites were included in the model, we found that the pre-departure rate of increase in calling intensity was lower (indicating a lack of consensus [13]) when jackdaws had spent a greater proportion of time calling during the night (estimate (s.e.) = −0.306 (0.148); *χ* ^2^ = 5.171, d.f. = 1, *p* = 0.023; figure 3*a*). Levels of background noise before the departure and all other terms had no significant effect (electronic supplementary material, table S6). We found the same result when excluding The Moor, with a negative effect of the proportion of time calling during the night being the only significant predictor (estimate (s.e.) = −0.294 (0.142); *χ* ^2^ = 4.820, d.f. = 1, *p* = 0.028; electronic supplementary material, table S7).

##### Prediction 4

The proportion of jackdaws that left together in the largest group departure ranged from 3.3% to 78.2% of individuals in the roost. Consistent with the analysis of pre-departure calling, we found that levels of overnight calling rather than noise levels prior to departure were linked to this temporal coordination of group departures. When jackdaws had spent a higher proportion of the night calling, the proportion of jackdaws that left the roost together in mass departures was lower (estimate (SE) = −0.556 (0.284); *χ* ^2^ = 5.504, d.f. = 1, *p* = 0.019; figure 3*b*). No other terms were significant in the model (electronic supplementary material, table S8).

##### Prediction 5

We found no significant effects of any predictors on the time taken for roost sites to empty (electronic supplementary material, table S9).

## 4. Discussion

This study provides evidence suggesting that anthropogenic disturbance, in particular noise pollution, may have important effects on overnight activity (a potential indicator of sleep disturbance) and collective behaviour in wild jackdaws. These results are consistent with previous research in captive animals and humans (e.g. [57,67,74,77]) and raise concerns over the impacts of sensory pollutants on animal vocal communication and collective group dynamics in an increasingly urbanized world.

### (a) Arrivals

We found that, in accordance with our predictions, jackdaws took longer to settle down and cease calling when they arrived at a roost site with higher background noise the hour before their arrival. This effect may come about if noise disturbs the jackdaws and causes them to call [92,109], particularly given that calling by one individual may then trigger calling by others (cf. [83]). Alternatively, jackdaws may exhibit increased levels of vocalizations if background noise acts as a cognitive distraction, inhibiting the processing of conspecific calls, even if calls can be heard [34,35]. For instance, when Caribbean hermit crabs (*Coenobita clypeatus*) were exposed to flashing lights alongside anthropogenic noise pollution, researchers could approach the crabs more closely than with noise alone, suggesting that anthropogenic disturbances can act as a distractor that can reduce responsiveness to cues [34]. It is also possible that the jackdaws’ higher levels of vocalizations represent a response to masking by noise pollution, similar to effects seen in other species [31,93,110–114]. For example, Wilson *et al*. [92] found that the number of singing Northern cardinals (*Cardinalis cardinalis*) increased almost fivefold in the presence of anthropogenic noise (drones being flown overhead), and there was also a significant increase in the number of contact and alarm calls produced during and after the presence of this noise disturbance.

Roost sites are hypothesized to be important for the sharing of social information in birds [115,116]. If signals are masked by high noise pollution upon arrival at roost sites, the exchange of information regarding predators and food availability could be detrimentally impacted [27,30]. Therefore, if anthropogenic noise reduces the fidelity of these signals, limiting the capacity of calls to be heard and information to be extracted by receivers [30], jackdaws may have to call for longer after arriving at the roost in order to share information effectively. If this is the case, it may explain why we only detected a correlation between background noise and post-arrival calling when the analysis included The Moor, as this busy town centre location may have been the only one of our roost sites where noise levels were sufficient to mask calls. Indeed, loud background noises at The Moor—in particular people talking loudly and traffic passing directly next to the roost trees—occasionally overlapped with the frequency of jackdaw calls, so it is reasonable to assume that noise pollution could mask jackdaw calls at this site. As levels of noise pollution correlate with site-level differences in artificial brightness, it is also possible that light pollution contributes to the prolonged post-arrival calling at The Moor.

When The Moor was excluded from the analysis, we also found evidence suggesting that larger groups settled down more quickly upon arrival. This result must be interpreted with caution as it may be confounded by site-level effects: for instance, the roost with the largest number of jackdaws (Gwennap) also covers the largest area, so individuals may be less likely to jostle noisily for position within the roost. It is also possible that faster rates of settling down reflect a group-level consensus, similar to patterns of inhibition seen in neural networks [117]. Future research linking patterns of calling to individual behaviour (perhaps using thermal imaging to capture the activities of individuals in the dark; cf. [118]) will be vital to evaluate these possibilities.

### (b) Night calling

As predicted, we also found evidence suggesting that as overnight background noise increased, jackdaws spent a greater proportion of the night calling. Given that roosting birds are known to sleep during the night [119,120] and that when birds are calling they must be awake, nocturnal calling activity may provide an indicator of sleep disturbance. Indeed, nocturnal activity triggered by anthropogenic disturbance has been associated with sleep debt in other studies—for example, in great tits, exposure to white light pollution caused a doubling of levels of overnight activity, with the most active individuals showing a greater reduction in oxalic acid concentrations [94], a key biomarker for sleep debt [121]. There is also evidence that the length and quality of sleep can be reduced by noise pollution in humans and other animals [57,70,94,122]. For example, Australian magpies exposed to noise across a 24 h period in laboratory conditions spent more time awake and were awoken more frequently [57]. Furthermore, mammals have been shown to demonstrate greater nocturnal activity in response to human activity [123]. Our results may reflect a similar pattern.

For the four sites where light level data were available, we found that, contrary to our expectation that light would cause disturbance, jackdaws called less during the night when light levels were higher. This could suggest that under lighter conditions, groups maintain contact visually, rather than relying on auditory communication (cf. [124]). We also found some evidence that night-time calling was associated with local weather conditions. In particular, it appeared that jackdaws called more during the night when the average windspeed was higher. Being buffeted by the wind and/or the sound of the wind itself or the movement of branches could potentially disturb sleeping or resting jackdaws and cause them to call. Higher wind speeds could also potentially trigger greater levels of wakefulness and vigilance, as seen in Eurasian oystercatchers (*Haematopus ostralegus* [125]). We also found evidence that jackdaws called less overnight when temperatures were higher. The reasons for this effect are unclear, though one possibility is that birds cluster more to retain heat in colder temperatures and so may be more likely to disturb each other and trigger calling. Important caveats to these results are that they were not robust in analyses excluding The Moor and that wind data were daily averages from nearby meteorological stations. In future work, analyses incorporating temporal changes in weather conditions within sites will be vital to understanding the fine-scale effects of local conditions.

Finally, similar to our arrival analyses, we found indications that jackdaws spent a greater proportion of the night calling when there were fewer individuals in the roost. The strength of evidence here is relatively weak as this association was only apparent in analyses of more rural sites, excluding The Moor, but it is possible that lower calling rates could reflect reduced perceptions of predation risk in larger groups [125–130]. This is reminiscent of findings in oystercatchers, where levels of overnight vigilance were lower when greater numbers of conspecifics were present [125].

### (c) Departures

Our results raise the intriguing possibility that collective departures from roosts are disrupted not directly by noise but indirectly through knock-on effects from night-time disturbance. Our previous work has shown that earlier mass group departures with greater temporal coordination are triggered by steep rises in calling intensity [13]. Here, we found that the rate of change in calling intensity was not directly associated with background noise levels in the previous hour, but rather with the proportion of time the jackdaws had spent calling overnight. Thus, rather than noise disrupting the establishment of a vocal consensus by masking calls, the disruption may be linked to the energetic expenditure of night-time calling and/or noise-induced sleep deprivation negatively impacting cognition and signal processing [28,29]. For instance, a reduction in sleep overnight could reduce the jackdaws’ capacity to process and relay acoustic signals, thereby also reducing the efficiency of their voting-like vocal consensus decision-making [13]. It is also possible that disruption to sleep could increase the discrepancy between individual interests, which may reduce the efficacy of consensus decisions. Overall, our results are consistent with growing evidence that sleep plays an important role in maintaining cognitive processes that facilitate acoustic communication and group dynamics [60,63,66].

Further suggestive evidence comes from our finding that higher levels of overnight calling were associated with reduced temporal coordination in collective departures. Again, we found no evidence for direct associations with background noise: the proportion of jackdaws that departed the roost together was not linked to morning noise levels but was lower if the jackdaws had spent more time calling during the night. Of course, our results are correlational and we have no direct measures of the birds' sleep, but our hope is that these findings will help to stimulate further research. Collective behaviours, including mass roost departures, are thought to have important fitness benefits by reducing predation risk, enabling social information transmission and increasing foraging efficiency [4,5,116]. If greater levels of disturbance disrupt sleep in wild animals, this could in turn reduce their ability to maintain cohesion and reap these benefits [38,80]. Our findings point towards important links between anthropogenic disturbance, calling, cognition and collective behaviour that warrant urgent further study. For field workers, one important next step will be to use playback experiments to test causal links between overnight disturbance and subsequent collective dynamics.

### (d) Future directions

It is important to acknowledge that due to the high correlation between noise and light pollution and the limited number of sites available, it is extremely challenging to disentangle the effects of noise and light pollution separately in this study. Our findings are consistent with the growing evidence that noise pollution affects acoustic communication [27,30,31], sleep [56,57] and collective behaviour [77–79]. However, we must be mindful that light pollution can confound apparent effects of noise in urban–rural gradients. To disentangle these effects, future work would benefit from selecting sites where noise and light disturbance are crossed where possible. Work will also require temporally and spatially fine-grained measures of light and noise in conjunction with concurrent records of vocalizations and behaviour. Technological advances make such work increasingly feasible: for instance, machine learning may allow us to accurately separate vocalizations from background noise [131], enabling us to test whether animals respond to fine-scale changes in light or noise profiles. Such careful observational studies, in combination with controlled experiments where noise or light is artificially manipulated (cf. [13,40,94]), will allow researchers to further unravel the effects of anthropogenic disturbance on sleep and collective behaviour.

Further research is also needed to understand the fitness impacts of the effects we have found. We have suggested that jackdaws may suffer negative consequences of disturbed communication, sleep and collective behaviour from anthropogenic disturbance, but it is possible that such effects may not all be deleterious. For example, higher light levels overnight could conceivably allow individuals to better assess the status and condition of roost mates, as suggested in great-tailed grackles (*Quiscalus mexicanus* [132]). Alternatively, birds may choose to roost in illuminated town centres as these sites are easier to find when birds are distributed over large areas [119]. Additionally, noise and light pollution may differentially affect group members depending on personality or sex-specific responses, as found in breeding great tits [133]. Research must, therefore, integrate population-level measures of disturbance with individual-based data to understand how variation in exposure to anthropogenic disturbance may influence fitness. For instance, future studies could investigate how personality type influences roosting behaviour and track the foraging and breeding success of birds across light and noise gradients.

## 5. Conclusions

Our study adds to the growing body of evidence that anthropogenic disturbance can affect the acoustic communication, behaviour and health of wildlife [26,27,134]. In particular, we highlight the arguably overlooked impacts of anthropogenic disturbance on social behaviour. As anthropogenic disturbance becomes more ubiquitous and widespread [135,136], animal communication is likely to suffer further impacts, leading to deleterious consequences in collective group dynamics. In some cases, these impacts may be exacerbated by the feedback loops inherent to collective systems, as responses to vocalizations cause disruption to cascade through groups. Such effects could have long-term consequences for wildlife populations [27]. While the relative effects of noise and light pollution were challenging to disentangle in this study, we hope that our findings highlight the importance of future research efforts linking lab and field studies, which will be vital to understand and mitigate impacts of human activities on acoustic communication and group dynamics.

## Data Availability

The datasets supporting this article have been uploaded in the Figshare digital repository [107]. Electronic supplementary material is available online [137].
